# Cavernous Hemangioma of the Tongue in an Adult

**DOI:** 10.7759/cureus.62800

**Published:** 2024-06-20

**Authors:** Yash Kalra, Akshita Goyal, Manu Babu, Paresh Chavan, Mayur Ingale

**Affiliations:** 1 Department of Otolaryngology, Head and Neck Surgery, Dr. D. Y. Patil Medical College, Hospital and Research Centre, Dr. D. Y. Patil Vidyapeeth, Pune, IND

**Keywords:** unusual presentations, tongue mass, benign vascular tumor, benign tumors, tongue hemangioma

## Abstract

Hemangiomas are benign tumors characterized by the proliferation of dilated blood vessels, typically capillaries and veins. They primarily occur in infancy and childhood, with the majority affecting the head and neck region. Oral hemangiomas, though relatively rare, can affect areas such as the lips, tongue, buccal mucosa, and palate. Despite their benign nature, managing vascular malformations is crucial due to potential functional loss and lifelong aesthetic concerns.

This case report involves a 76-year-old woman presenting with a soft reddish-blue mass on the dorsal aspect of her tongue, causing functional impairment. While various treatment options exist for oral vascular malformations, including sclerotherapy and cryosurgery, surgical excision was chosen in this case, considering the patient's age and the associated risks of the condition.

## Introduction

Hemangiomas are benign tumors formed from enlarged blood vessels, typically veins and capillaries, within a specific area of submucosal connective tissue. The terms "hemangioma" and "vascular malformation" have often been used alternately, causing notable confusion in clinical practice and literature. According to Mulliken and Glowacki's 1982 classification system, "hemangiomas" are considered true neoplasms characterized by increased proliferation and turnover of endothelial cells. In contrast, "vascular malformations" are localized anomalies resulting from abnormalities in vascular development with normal rates of cell turnover [1].

The occurrence of oral hemangiomas in the head and neck area is prevalent, ranging from 60% to 70%. However, they are relatively uncommon on the tongue, and when present, they tend to be more frequent on the underside (ventral surface) of the tongue. There is a notable female predominance, with a ratio of 3:1 compared to males. Typically viewed as an infancy-related condition, oral hemangiomas affect up to 6.4% of infants, although there is limited data available to estimate their prevalence in older age groups [1]. Usually, oral hemangiomas become clinically apparent a few weeks after birth and then grow rapidly, but in the majority of cases, they eventually undergo slow involution to near spontaneous resolution [2]. Generally, oral hemangiomas become noticeable clinically a few weeks after birth and tend to grow rapidly during this early phase. However, in the majority of cases, they eventually undergo a gradual involution process, leading to near-spontaneous resolution over time [3]. We describe a case with a rare and unusual presentation of a cavernous hemangioma of the tongue in a 76-year-old female and its management.

## Case presentation

A 76-year-old female patient presented with the complaint of a swelling of approximately the size of a grape on the tip of the tongue for four months. The swelling was insidious in onset and non-progressive in size. The patient also complained of pain over the swelling while speaking, which had aggravated in the past fifteen days. There was no history of bleeding or discharge from the swelling. The patient does not complain of any trauma to the tongue or difficulty in mastication or swallowing. No other significant positive history was present. The patient did not have any relevant past, personal, or surgical history.

**Figure 1:**
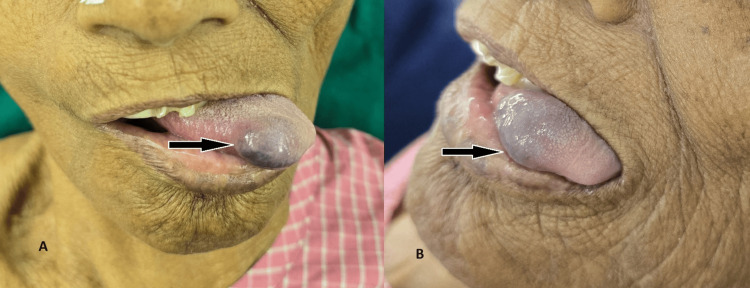
Preoperative images showing the hemangioma over the tongue A: Central profile with the black arrow showing bluish ovoid swelling approximately 2 × 1 cm in size over the right dorsal aspect of the tongue tip B: Left lateral profile with the black arrow showing bluish ovoid swelling as described above

On clinical examination of the oral cavity, a solitary blue-colored swelling of approximately 2 × 1 cm was present over the right lateral aspect of the dorsal part of the tip of the tongue. The swelling was sessile and compressible, with a smooth surface and diffuse margins. There was no ulceration over the swelling and no attachment to the underlying muscles. The swelling did not bleed on touch, was minimally tender, blanched on application of pressure, and was soft in consistency. There was no restriction on tongue movements. No pulsations were present on palpation (Figure 1).

Based on the clinical history and examination, a differential diagnosis of lymphangioma, hemangioma, and pyogenic granuloma was made. All routine investigations of the patient were within normal limits (Table 1).

**Table 1:** Routine preoperative blood parameters g/dL - grams per deciliter; HbsAg - hepatitis B surface antigen; HCV - hepatitis C virus; HIV - human immunodeficiency virus; IU/mL - international units per milliliter; mg/dL - milligrams per deciliter; mm/hour - millimeter per hour; mmol/L - millimoles per liter; S/CO - signal-to-cut-off ratio; SGOT - serum glutamic oxaloacetic transaminase; SGPT - serum glutamic pyruvic transaminase; /µL - per microliter; U/L - units per liter

Name	Values	Biological references
Hemoglobin (g/dL)	11.4	11.6-15.0
Total leucocyte count (/µL)	10,100	4,000-11,000
Platelet count (/µL)	290,000	150,000-410,000
Blood group	A positive	-
Total bilirubin (mg/dL)	0.02	0.22-1.2
SGOT (U/Lt)	22	08 to 43
SGPT (U/Lt)	7	07 to 45
Erythrocyte sedimentation rate (mm/hour)	22	Up to 30 mm/hour
Random blood sugar (mg/dL)	97	<200 mg/dL
Sodium(mmol/L)	142	136-145
Potassium (mmol/L)	3.52	3.5-5.1
HIV (S/CO)	Non-reactive	-
HBsAg (IU/mL)	Non-reactive	-
HCV (S/CO)	Non-reactive	-
Prothrombin time in seconds	11.5	10.24-12.71
International normalized ratio	0.97	0.85-1.15

**Figure 2:**
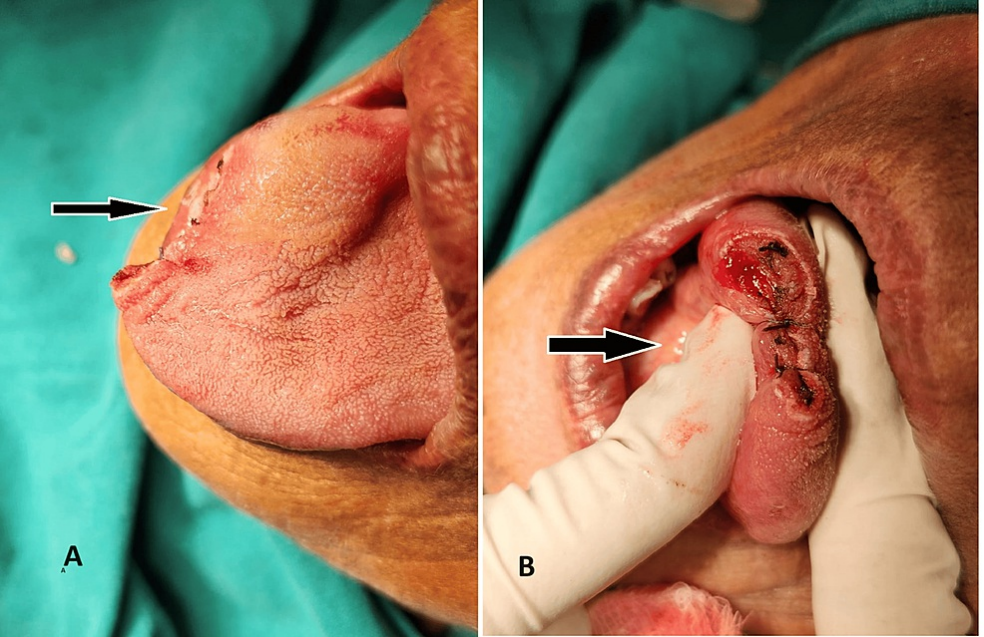
Intraoperative pictures showing the tongue post-excision A: Tongue after excision of hemangioma B: Tongue after primary closure using Vicryl 3-0 absorbable sutures

The patient was posted for surgical excision under local anesthesia after obtaining relevant consent (Figure 2).

In this case, biopsy and fine needle aspiration cytology were not indicated as a vascular mass has a high propensity to bleed uncontrollably. Color Doppler and computed tomography angiography/magnetic resonance imaging can be performed in cases with large-sized hemangiomas to identify the feeder vessel. Preoperative embolization can then be done if required to reduce intraoperative complications.

**Figure 3:**
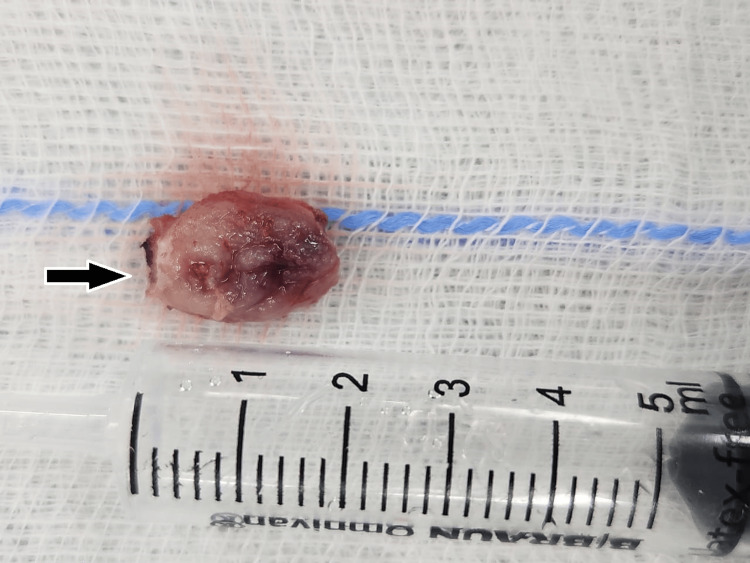
Image of the postoperative gross specimen

The mass was surgically excised and sent for histopathological examination. The gross specimen was oval, soft, and firm in consistency with an uneven surface (Figure 3).

**Figure 4:**
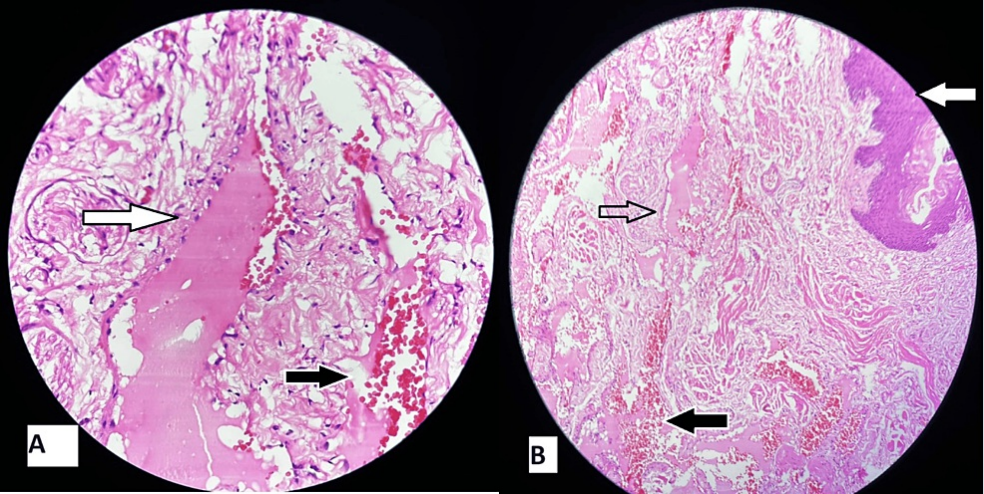
Histopathological images of the tongue specimen A: White solid fill arrow - marking the cavernous space; black solid fill arrow - marking the red blood cells B: White solid fill arrow - marking the stratified squamous lining epithelium; black arrow without fill - marking the cavernous space; black solid fill arrow - marking the red blood cells

The histopathological examination revealed stratified squamous lining epithelium. Sub epithelium showed cavernous spaces lined by endothelial cells and filled by red blood cells. No evidence of atypia or malignancy was present (Figure 4). A definitive diagnosis of cavernous hemangioma was made.

**Figure 5:**
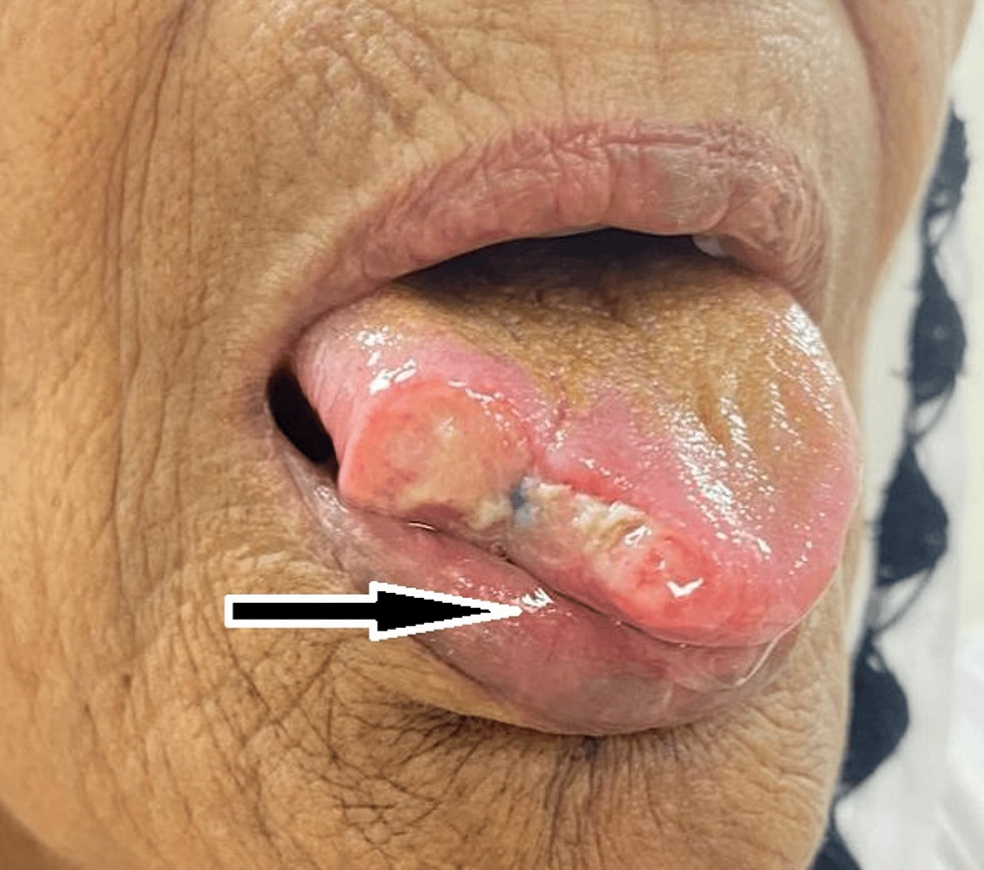
Patient's tongue on follow-up Black arrow showing the healed wound site

Postoperatively, the patient had no complications during wound healing, and complete recovery was seen within one month of surgery (Figure 5). The patient is under regular follow-up, and there have been no signs of recurrence.

## Discussion

Hemangioma, derived from Greek roots meaning "blood vessel tumor," is defined as a benign growth of dilated blood vessels. It can present in various forms, such as port-wine stains, strawberry hemangiomas, and salmon patches. The exact cause of oral hemangiomas is unclear, but genetic mutations, hormonal influences, and embolic events may play significant roles in their development. The life cycle of a hemangioma typically involves three stages: endothelial cell proliferation, rapid growth, and spontaneous involution. Endothelial cell proliferation marks the active growth phase of a hemangioma, during which there is an overexpression of cytokines and angiogenesis-related molecules like fibroblast growth factor 2 (FGF-2), vascular endothelial growth factor A (VEGF-A), and matrix metalloproteinases. In the involution phase, mesenchymal stem cells differentiate into adipocytes, and capillary lumens undergo apoptosis, leading to the gradual resolution of the lesion [4]. In 80% of the cases, hemangiomas undergo involution and spontaneous resolution and do not warrant the need for treatment, but in 10-20% of the cases where resolution does not take place, the patient may experience varied symptoms depending on the location of the hemangioma requiring treatment.

The tongue, being a mobile and muscular organ, makes it very vulnerable to injury, leading to the risk of profuse bleeding, and patients may present with spontaneous hemorrhage that resolves on its own. The terminology used previously categorizes oral hemangiomas into capillary, cavernous, or mixed types, but a more accurate classification considers the depth of the lesion as superficial, deep, or compound hemangiomas. Superficial hemangiomas appear as bright red macular or papular masses originating from the papillary dermis, previously known as strawberry hemangiomas. Deep hemangiomas manifest as relatively colorless or bluish masses arising from the reticular dermis or subcutaneous tissues. A lesion exhibiting a thrill or bruit may indicate a specialized vascular malformation known as arteriovenous hemangioma. There are various syndromes associated with vascular malformations, including Osler-Weber-Rendu syndrome (hereditary hemorrhagic telangiectasia), Sturge-Weber syndrome, and blue rubber bleb nevus syndrome. [5]. Diagnosing oral hemangiomas is generally straightforward and based on patient history and clinical examination findings. However, in some cases, contrast-enhanced MRI or angiography may be necessary. These imaging techniques help determine the depth of the mass and provide detailed information about the vascularization of larger hemangiomas, aiding in treatment planning and management decisions. [6].

Treatment for hemangiomas can vary depending on factors such as the patient's age and the location and size of the lesion. Options typically include the following: medical therapy, laser therapy, and surgical resection. Medical therapy can involve medications like propranolol (a beta-blocker) or systemic steroids to reduce the size and symptoms of the hemangioma. Laser therapy, such as laser photocoagulation, can be used to shrink or remove superficial hemangiomas. In some cases, particularly for larger or deeper hemangiomas, surgical removal may be considered. The choice of treatment is tailored to each individual case, taking into account the specific characteristics and potential risks associated with the hemangioma. [7]. Systemic corticosteroids have their own systemic side effects, while according to studies, only 30% of the population responds to steroids [8]. In cases of severe symptoms like airway obstruction, dysphagia, and bleeding, aggressive treatments are indicated. A wider surgical approach is employed for lesions enormous in size, while small lesions can be excised easily [9]. Sclerotherapy is a newer modality of treatment that includes injecting one of the major feeding vessels with a sclerosing agent, leading to endothelial damage and obliteration of the lumen. Even though sclerotherapy has been shown to have favorable outcomes, it carries with it the risk of thrombosis with embolization, especially in elderly patients. Each treatment method has advantages and disadvantages. Conservative methods give rise to recurrences, while aggressive treatment may cause lingual tissue loss and major functional disability. Multiple treatment modalities can be combined to manage the disease as well [10]. Radiation therapy is known to shrink hemangiomas and is considered one of the available treatment options. However, it also severely atrophies the treated area's tissues, particularly the skin. This makes it an unfavorable option, as it can cause cancer in later years [11]. Plasma knife surgery was used for the excision of a hemangioma of the tongue by Kutluhan [12].

Since histopathological reporting is the only definitive way of confirming the diagnosis, the treating physician is required to be well-versed in the clinical presentation and findings of the disease, as well as various treatment modalities, to avoid unnecessary investigations and to choose the appropriate treatment protocol while managing patients.

## Conclusions

Hemangioma is among the most common soft tissue tumors seen in infants and children. The case discussed involves an elderly woman with a small cavernous hemangioma located on the tip of her tongue, a rare occurrence in this age group. Due to the brief history of presentation, the small size of the lesion, and its accessible location, the decision was made to surgically remove it. Early detection and intervention are crucial to preventing complications. Treatment choice depends on factors such as the lesion's location, size, and the patient's age. Current literature suggests promising outcomes with sclerotherapy and cryosurgery, although further research in this area is needed. Therefore, considering our patient's age, the location, and the size of the lesion, surgical excision was deemed the most appropriate management option.
